# Vascular Hyperactivity in the Rat Renal Aorta Participates in the Association between Immune Complex-Mediated Glomerulonephritis and Systemic Hypertension

**DOI:** 10.3390/ijerph15061164

**Published:** 2018-06-03

**Authors:** Israel Pérez-Torres, Bernardo Moguel-González, Elizabeth Soria-Castro, Verónica Guarner-Lans, María del Carmen Avila-Casado, Teresa Imelda Fortoul Vander Goes

**Affiliations:** 1 Departamento de Patología, Instituto Nacional de Cardiología “Ignacio Chávez”, Ciudad de México 14080, México; bernardomoguel@hotmail.com (B.M.-G.); elizabethsoria824@gmail.com (E.S.-C.); carmen.avila-casado@uhn.ca (M.d.C.A.-C.);; 2 Departamento de Fisiología Instituto Nacional de Cardiología “Ignacio Chávez”, Ciudad de México 14080, México; gualanv@yahoo.com; 3 Departamento de Biología Celular y Tisular, Facultad de Medicina, Universidad Nacional Autónoma de México, Ciudad de México 04510, México; fortoul@unam.mx

**Keywords:** glomerulonephritis, hypertension, reactivity vascular, eNOS, iNOS, immune complex

## Abstract

*Introduction*: systemic hypertension (SH) involving endothelial dysfunction contributes to immune complex-mediated glomerulonephritis (ICGN). Objective, we demonstrate a relationship between ICGN and SH by analyzing vascular reactivity in renal aortic rings. *Methods*: 48 male Wistar rats were divided into four groups: (a) control (C); (b) injected with bovine serum albumin (BSA); (c) receiving 200 mg/L NAME (an analog of arginine that inhibits NO production) in drinking water; and (d) receiving BSA and 200 mg/L NAME. Rats were pre-immunized subcutaneously with BSA and Freund’s adjuvant. After 10 days, groups (b) and (c) received 1 mg/mL of BSA in saline intravenous (IV) daily for 35 days. The urine of 24 h was measured at days 0, 15, 30 and 45. *Results*: vascular reactivity to norepinephrine (NE), acetylcholine (Ach) and NAME were tested. Creatinine clearance, vasodilatation, eNOS and elastic fibers were diminished (*p* ≤ 0.001). Blood pressure, vasoconstriction, iNOS were increased, and glomerular alterations were observed in groups (b), (c) and (d) when compared to group (a) (*p* ≤ 0.001). *Conclusions:* SH contributes to the development of progressive renal disease in ICGN. Alterations of the vascular reactivity are mediated by the endothelium in the renal aorta. Thus, the endothelium plays a determinant role in the production of vasoactive substances such as NO during this process.

## 1. Introduction

Renal diseases represent a serious worldwide problem and their study in experimental models constitutes an important tool to understand their pathogenesis and their natural evolution [1]. It is nowadays accepted that the majority of glomerulonephritis occurring in humans has an immunologic origin [2]. This hypothesis is supported by the presence of deposits of immune complexes with complement protein in renal biopsies and by the presence of inflammatory cells and electron-dense immune deposits [3]. In addition, clinical and laboratory findings have demonstrated the presence of immune alterations and the efficacy of immunosuppressive treatments [4]. Although these observations could be incidental, studies in experimental animal models of glomerulonephritis that simulate glomerulonephritis in humans have proven the role played by the immunologic processes in the pathogenesis of the illness [5]. The first experimental model was created by Dixon [6]. Most of the active models of the illness use sharp and chronic serum and have been carried out by repeating injection of protein substances that act as immunogens eliciting antibody formation. During the active phase of elimination of the circulating antigen, there is formation of antigen–antibody complexes that are deposited on the walls of arterioles and capillaries [7]. The sequential administration of antigens (cationic or anionic macromolecules and specific antibodies) in these models [7,8] shows a pattern of membrane damage with the presence of immune complexes located in the sub-epithelium [8]. Besides, factors other than the deposit of the immune complexes exist, which contribute to progressive renal deterioration leading to the renal terminal phase of the illness in patients. Among these, systemic hypertension (SH) plays an important role [9].

SH is a multifactorial pathology influenced to a large extent by environmental factors. Together with genetic predisposition, it will cause short- and long-term disorders in organs and systems that result in a decrease in the survival rate of patients [10]. Due to the important role of the kidney in the long-term control of SH, the development of various degrees of arterial SH is one of the most frequent manifestations of chronic renal failure in patients [11]. Kidney artery involvement in SH involves loss of renal mass which aggravates the underlying hypertension [12].

The immune complex-mediated glomerulonephritis (ICGN) and SH can be related by two mechanisms: (a) SH may lead to renal progression damage in a previously established glomerulopathy, and/or (b) SH may determine the deposition of immune complexes in the kidney [2,13].

The relationship between ICGN and SH has not been fully established and therefore, the goal of this investigation was to demonstrate the relationship between ICGN and SH by the analysis of vascular reactivity in rat renal artery rings.

## 2. Material and Methods

### 2.1. Animal Treatment

Experiments in animals were approved by the Laboratory Animal Care Committee of our institution and conducted in compliance with the Guide for the Care and Use of Laboratory Animals. Rats were maintained under a 12-h dark–light cycle and controlled temperature of 22 ± 2 °C. Water and rodent commercial food (23% of crude protein, 4.5% of crude fat, 8% of ashes, and 2.5% of added minerals) were given to the animals ad libitum. 48 male Wistar rats (300–350 g) were divided in 4 groups (*n* = 12 each): (a) control group (c) which received no injections, (b) rats that received bovine serum albumin (BSA), (c) rats that received 200 mg/L of NAME (nitro arginine-L-methyl ester) in drinking water and (d) rats with BSA and 200 mg/L of NAME. BSA and BSA + NAME groups were subcutaneously pre-immunized by injections at the tail’s base of 1 mg of n-BSA in complete Freund’s adjuvant containing 5 mg/mL H37RA *Mycobacterium tuberculosis* (Difco Laboratories, Detroit, MI, USA). After a 10 day time period, groups (b) and (d) received an IV injection of 1 mg/mL of BSA in saline every day for 35 days. 

### 2.2. Systolic Blood Pressure

At the end of the treatment, blood pressure (BP) was measured by a tail-cuff attached to a pneumatic pulse transducer method (Narco Bio-Systems Inc., Houston, TX, USA), in accordance with the method described by Pérez-Torres [14].

### 2.3. Proteinuria and Glomerular Filtration

At the end of the treatment and before sacrifice, the animals were placed in metabolic cages (Nalgene); urine was filtered and collected for 24 h. Albuminuria was measured using bromocresol green reagent. This technique is specific for the quantification of albumin in urine [15]. Serum and urine creatinine (SCr and UCr, respectively) were measured by the Jaffe method [16]. Creatinine clearance (CrCl) was calculated according to the following formula: [UCr]/[SCr] × urinary volume/time. At the end of the treatments, animals were decapitated and blood was collected. Blood samples were centrifuged at 850 *g* for 20 min at 4 °C and serum was recovered. Serum was used for the measurement of SCr. 

### 2.4. Histological Preparation

The right kidney and a section of renal aorta were washed in 0.9% NaCl for 30 s immediately before the tissues were fixed by immersion in phosphate buffer with 10% formalin (pH 7.4) for 24 h. The histological sections of the kidney were processed according to conventional histological procedures by hematoxilin–eosin stain. An average of 30 glomeruli per level of section in each sample was examined. The presence of immune deposits was evaluated by direct immunofluorescence. Frozen renal tissue was stained with -bound monoclonal antibodies against rat IgG-FICT (Caltag). 30 glomeruli were examined per case. Fluorescence intensity was evaluated in a semi-quantitative form in a scale of 0 to 3+ (0, negative, 1+, weak, 2+, moderate, and 3+, strong). In addition, renal aorta sections were processed with Weigert’s method for elastic fibers [17], and immune histochemistry with eNOS and iNOS antibodies (Santa Cruz Biotechnology, Santa Cruz, CA, USA) at a final dilution of 1:2000. Histological sections were analyzed using a Carl Zeiss light microscope (Carl Zeiss, West Germany, Germany) (63,300 model) equipped with a Tucsen (9 megapixels) digital camera with software TSview 7.1 (Tucsen Imaging Technology Co., Ltd. Chuo, Japan, at a 40× magnification. The photomicrographs were analyzed by densitometry using Sigma Scan Pro 5 Image Analysis software Systat Software Inc. San Jose, California, CA, USA. The density values are expressed as pixel units.

### 2.5. Vascular Reactivity

After decapitation, the renal aorta was dissected and sectioned in rings of 2 mm that were suspended by metallic hooks in 5 mL glass chambers for isolated organs. One of the hooks was attached to the bottom of the chamber and the other to a tension transducer and a model 79D Grass polygraph with a recorder. The chamber contained Krebs solution (118 NaCl, 1.2 KH_2_PO_4_, 24 NaHCO_3_, 4.7 KCl, 1.2 MgSO_4_ (7 H_2_O), 2.5 CaCl_2_ (2H_2_O) 4.5 glucose, mM), pH 7.4, which was constantly bubbled in a gas mixture of 95% O_2_ and 5% CO_2_ and kept at a constant temperature of 37 °C. A basal tension of 2 g was applied to the rings. The aortic preparations were allowed to stabilize in the Krebs solution for one hour with two solution changes every 30 min. Dose-response curves to acetylcholine (Ach) were obtained; the ring’s contraction was induced initially with 2 × 10^−7^ M NE (norepinephrine) and then 2 × Ach was added in concentrations from 2 × 10^−9^ to 10^−5^ M accumulatively. This method has been previously described by Pérez-Torres [18]. For the contraction curves NE was added at increasing concentrations; 2 × 10^−9^ to 2 × 10^−5^ M; when the maximal contraction response curve was reached for each concentration, the next concentration was added. After obtaining each series of contraction or relaxation curves, the aortic rings were washed three times with Krebs solution and allowed to recuperate for 30 min [18].

### 2.6. L-NAME

The renal aorta preparations were incubated with the NO synthase inhibitor L-NAME (300 µM) (Sigma-Aldrich., Co. St Louis, Missouri, MO, USA) for 10 min, then 2 × 10^−7^ M NE (Sigma-Aldrich., Co. St Louis, Missouri, MO, USA) was added and the maximal contraction was registered. The information was added.

### 2.7. Statistical Analysis

Statistical analysis and the figures were obtained with the Sigma Plot version 12.3 software (SigmaPlot, Jandel Corporation, San José, CA, USA). The data are presented as mean ± standard error. Statistical significance was determined by Student’s *t*-test; differences were considered statistically significant when *p* ≤ 0.05.

## 3. Results

### 3.1. Blood Pressure

Table 1 shows a significant decrease in the BP in the C group (97 ± 2 mmHg, *p* = 0.001) in comparison to BSA and NAME groups (126 ± 2 mmHg, 147 ± 1 mmHg, respectively). The BSA + NAME group was significantly higher (173 ± 5 mmHg) than the BSA and NAME groups (*p* = 0.001).

### 3.2. Proteinuria and Glomerular Filtration

Table 1 shows the proteinuria level patterns during the experimental time in the four groups of rats. The BSA and the NAME groups showed a statistically significant difference in the level of protein excretion during the time of evolution (50.9 ± 10 mg/24 h and 162.3 ± 31 mg/24 h, *p* = 0.05 and *p* = 0.001, respectively) in comparison with C (29.8 ± 3 mg/24 h). In the BSA + NAME group, the proteinuria was significantly increased to 178.6 ± 23 mg/24 h (*p* = 0.001) in comparison with BSA group. Likewise, Table 1 shows that the SCr level of the BSA and NAME groups were significantly increased (0.95 ± 0.10 µg/dL and 1.18 ± 0.12 µg/dL, respectively) in comparison to the C group (0.68 ± 0.05 µg/dL, *p* = 0.001). In the BSA + NAME group, this increase was greater than in the BSA group (1.35 ± 0.09 µg/dL). Besides, Table 1 shows that UCr levels were increased in the BSA and NAME groups (0.52 ± 0.19 mg/dL and 0.76 ± 0.06 mg/dL, respectively) when compared to the C group (1.06 ± 0.08 mg/dL, *p* = 0.01). The BSA + NAME group (0.67 ± 0.36 mg/dL) did not show a significant difference with the BSA group. Moreover, Table 1 shows the CrCl was significantly diminished in the BSA and NAME groups (0.0073 ± 0.0006 mL/min and 0.0066 ± 0.0004 mL/min, *p* = 0.01 and *p* = 0.001, respectively) in comparison with the C group. The BSA + NAME group showed a statistically significant decrease in the CrCl when compared to the BSA group (0.0051 ± 0.0004 mL/min, *p* = 0.05).

### 3.3. Vascular Reactivity

#### 3.3.1. Vasoconstriction

**Figure 1:**
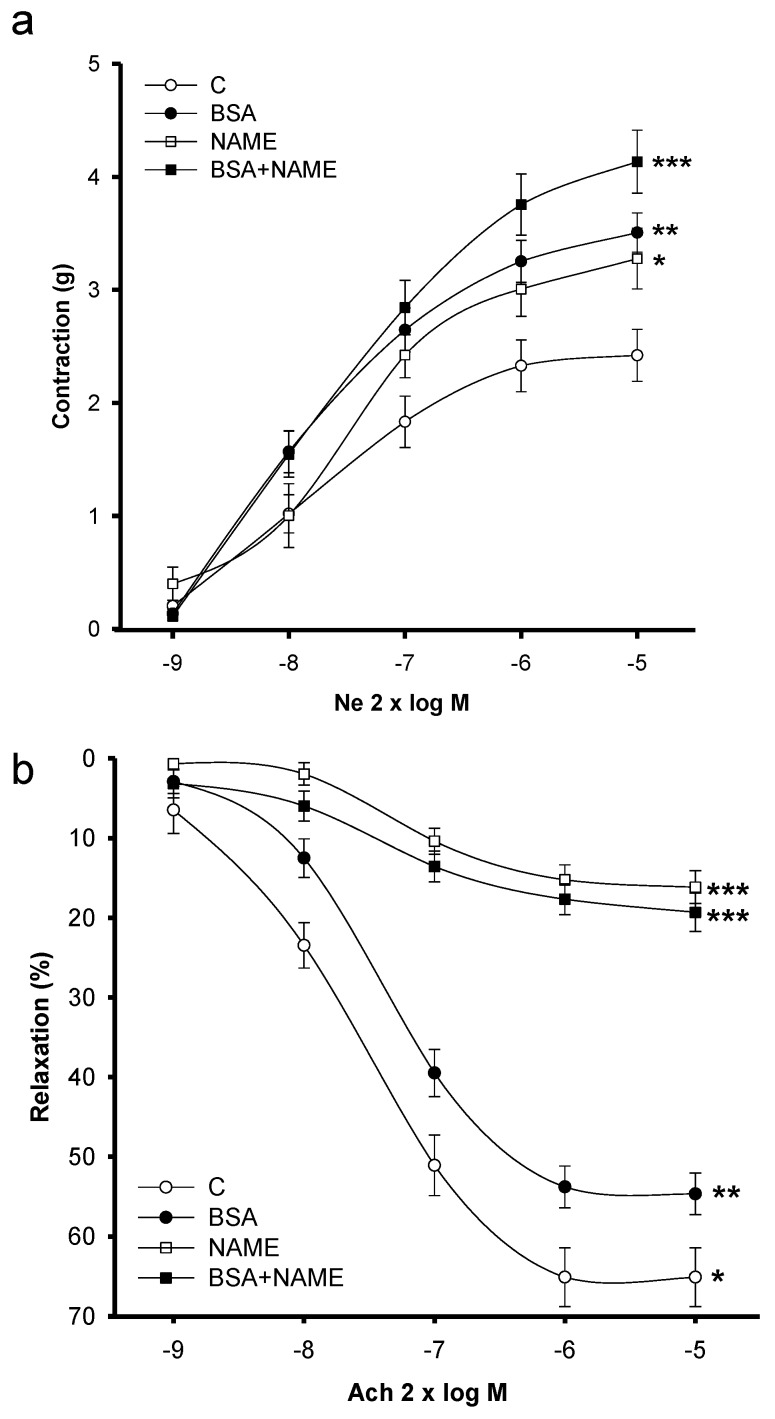
(**a**) Shows the vascular reactivity in aortic renal level response to 2 × 10^−7^ NE; (**b**) shows the aortic renal level response to 2 × 10^−7^ Ach. Data are means ± SE, *n* = 12 in each group. Significantly different from * C vs. BSA *p* = 0.05, ** C vs. NAME and BSA vs. BSA + NAME *p* = 0.01, *** C vs. BSA and NAME *p* = 0.001. NE: norepinephrine. Ach: acetylcholine, log M: logarithm of Molarity.

Figure 1A shows the NE-induced vasoconstriction responses of the BSA and NAME aortic rings. Vasoconstriction was higher in both groups in comparison with that of C rings (*p* = 0.01). The vasoconstriction in the BSA + NAME rat aortic rings showed a significant increase in comparison with BSA and NAME groups (*p* = 0.001).

#### 3.3.2. Vasodilatation

Figure 1B shows the Ach-induced vasodilation responses in endothelium-intact aortic rings in the four groups of rats. Cumulative concentration-response curves to Ach (2 × 10^−9^ to 2 × 10^−5^ M) were generated in rings contracted with NE (2 × 10^−7^ M). Relaxation to Ach (2 × 10^−9^ to 2 × 10^−5^ M doses) was significantly decreased in aortic rings from BSA- and NAME-treated rats (*p* = 0.01 and *p* = 0.001, respectively) in comparison to C rings. The vasodilatation observed in the BSA + NAME rat aortic rings showed a significant decrease in comparison with the BSA group (*p* = 0.001).

#### 3.3.3. Effect of L-NAME

**Figure 2:**
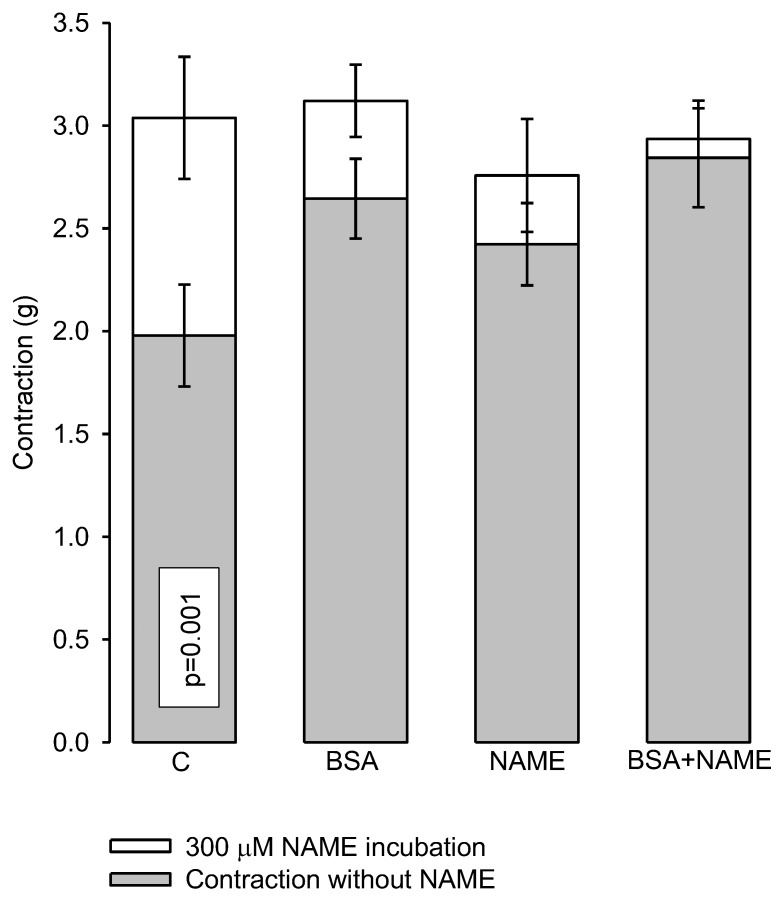
Maximum vascular response to NE in renal aortic rings in the presence of 300 µM L-NAME. Data are means ± SE, *n* = 12 in each group. Significantly different from C with NAME vs. C without NAME.

Aortic rings in the presence of 300 µM L-NAME (a competitive antagonist of the endothelial nitric oxide synthase (eNOS) (Figure 2)) showed a significant increase in the vasoconstrictor response to NE (2 × 10^−7^ M) in aortic rings of the C group in comparison to rings contracted without the presence of the antagonist (*p* = 0.001). In the BSA, NAME and BSA + NAME groups, the increase in the vasoconstriction response to the presence of the antagonist was not observed.

#### 3.3.4. Histological Characteristics (Elastic Fibers) in Renal Artery

**Figure 3:**
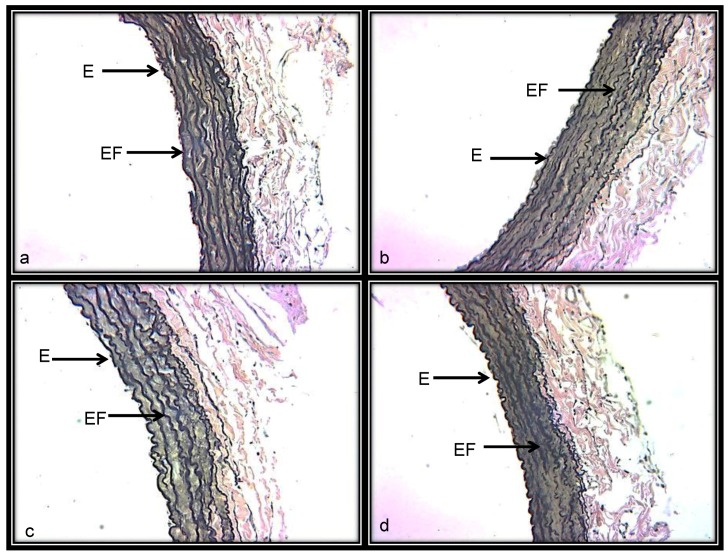
Representative photomicrograph of the aortic medial layer stained with Weigert’s method. (**a**) = Control; (**b**) = BSA; (**c**) = NAME; and (**d**) = BSA + NAME. Abbreviations: E = endothelium and EF = elastic fibers.

**Figure 4:**
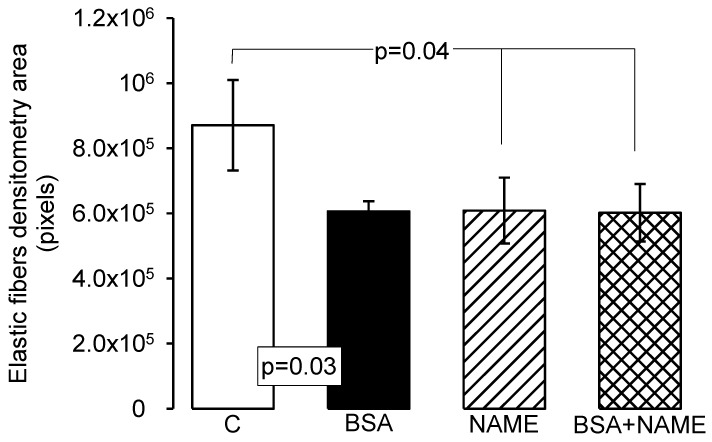
Photometric density analysis from marked area by Weigert’s method, the elastic fibers highlighted in black, in the four experimental groups. Data are means ± SE, *n* = 12 in each group.

Figure 3a shows a representative photomicrograph of the aortic medial layer stained with Weigert’s method. In the renal aortas from the C group, elastic fibers were preserved without rupture and alternated with nuclei of the vascular smooth muscle cells. Figure 3b,c shows increases in collagen between the thickened elastic fibers and the presence of fiber breakage in some areas in response to BSA and NAME treatments. Figure 3d corresponds to renal aortas from the BSA + NAME-treated group, and in it, bundles of elastic rolling fibers thickened and fragmented in black color that alternate with collagen fibers are present. The photometric density analysis of areas shows a decrease in elastic fibers in aortas from the BSA-, NAME- and BSA + NAME-treated groups in comparison to the C group (*p* = 0.03 and *p* = 0.04, respectively, Figure 4).

**Figure 5:**
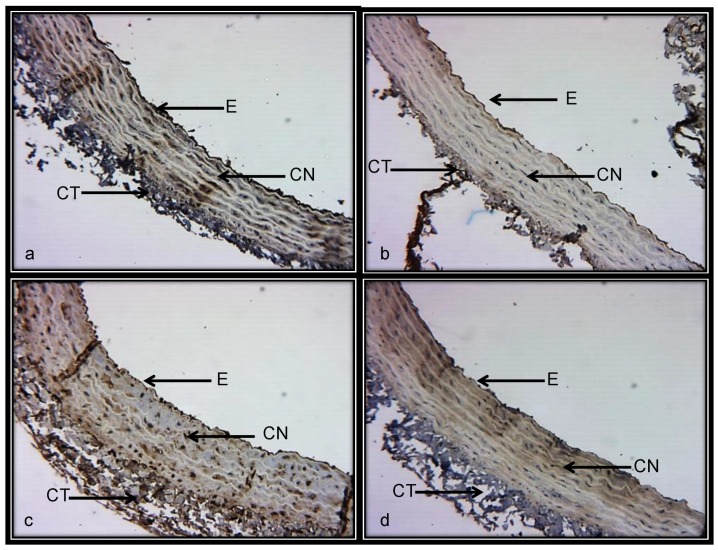
Representative photomicrograph of the aortic medial layer marked with eNOS antibody. (**a**) = Control; (**b**) = BSA; (**c**) = NAME; and (**d**) = BSA + NAME. Abbreviations: E = endothelium, CT = connective tissue and CN = cell nucleus.

**Figure 6:**
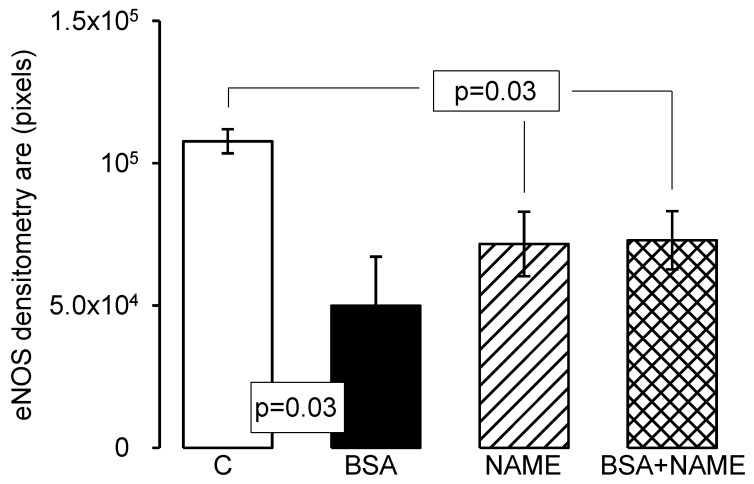
Photometric density analysis from marked area by eNOS antibody in the four experimental groups. Data are means ± SE, *n* = 12 in each group.

Figure 5a–d shows representative photomicrographs of the renal aorta segment in the C, BSA, NAME and BSA + NAME groups, respectively, marked with an eNOS antibody. The endothelium stained in brown corresponds to the intima area in which eNOS is located. In the C group, an intact endothelium without rupture is observed, however, in the renal aortas from BSA-, NAME- and BSA + NAME-treated groups, ruptured and scarce endothelium is observed. The photometric density analysis shows a significantly decreased area of eNOS in the three treated groups in comparison to the C group (*p* = 0.03, Figure 6). 

**Figure 7:**
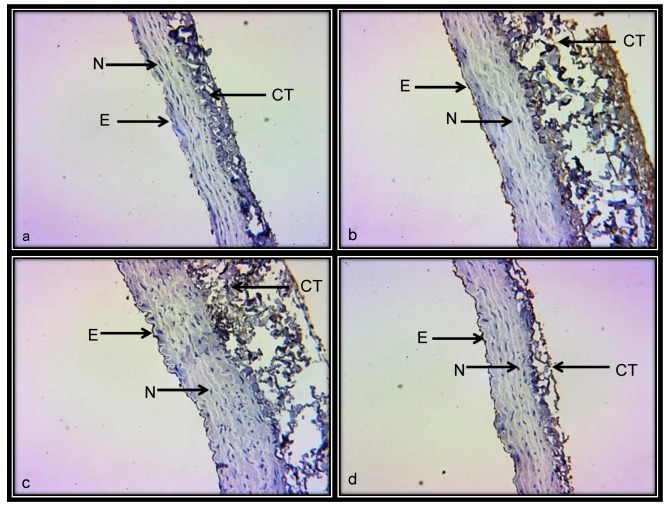
Representative photomicrograph of the aortic medial layer marked with iNOS antibody. (**a**) = Control; (**b**) = BSA; (**c**) = NAME; and (**d**) = BSA + NAME. Abbreviations: E = endothelium, CT = connective tissue and CN = cell nucleus.

**Figure 8:**
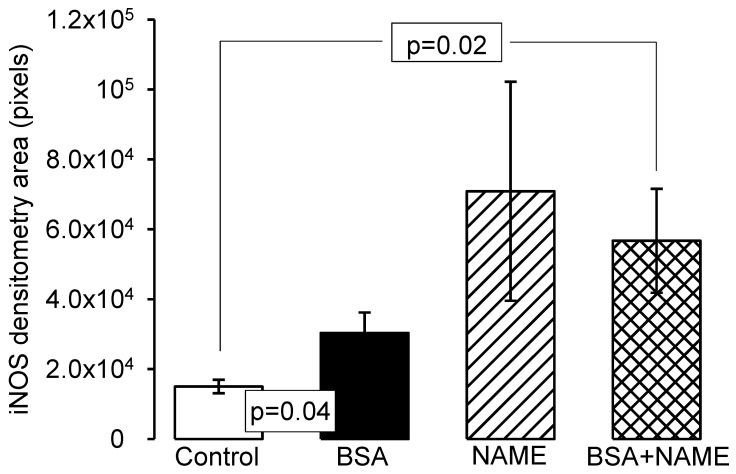
Photometric density analysis from marked area by iNOS antibody in the four experimental groups. Data are means ± SE, *n* = 12 in each group.

Figure 7a–d shows representative photomicrographs of the renal aorta segment in C, BSA, NAME and BSA + NAME groups, respectively, marked with iNOS antibody. In C group, the labeling of the iNOS is scarce. However, in the BSA, NAME and BSA + NAME groups, the labeling on the endothelium is intense when compared to that in the C group. The photometric density analysis showed a significantly increased iNOS area in BSA, NAME and BSA + NAME groups in comparison to C group (*p* = 0.04 and *p* = 0.02, respectively, Figure 8).

#### 3.3.5. Histological Features in Kidney

**Figure 9:**
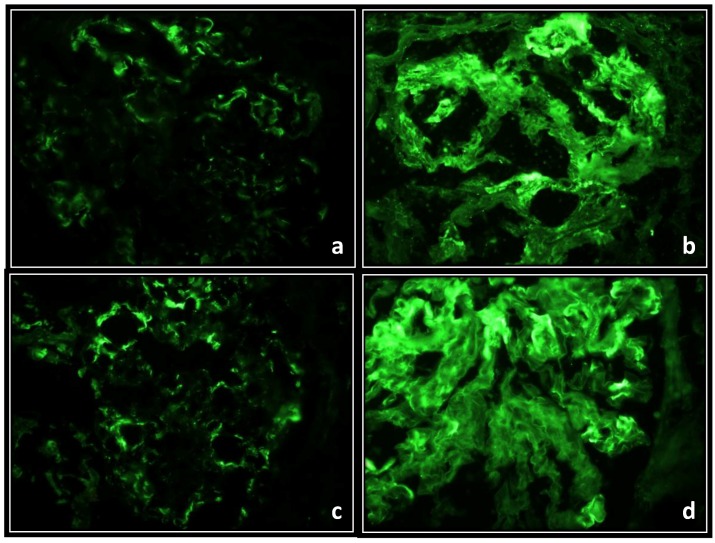
Representative direct immunofluorescence for IgG in glomeruli of the experimental groups to 40×, where in (**a**), which represents group C, some capillary loops with a weak signal can be observed; (**b**), which represents group BSA, all capillary loops and the mesangial zone show a strong signal; (**c**), which represents group NAME, the intensity in the capillary loops is slightly stronger than in group C; and (**d**), which represents group BSA + NAME, the capillary loops are thickened by immune-complex deposits and the observed signal is very strong in them and in the mesangial zone; capillary lumen of the capillary is diminished or almost absent.

Direct immunofluorescence against rat IgG was negative for the presence of immune-complex rats in the C and NAME groups (Figure 9a,c, respectively). However, in the BSA and BSA + NAME groups, direct immunofluorescence showed IgG-positive immune-complex deposition (Figure 9b,d, respectively).

**Figure 10:**
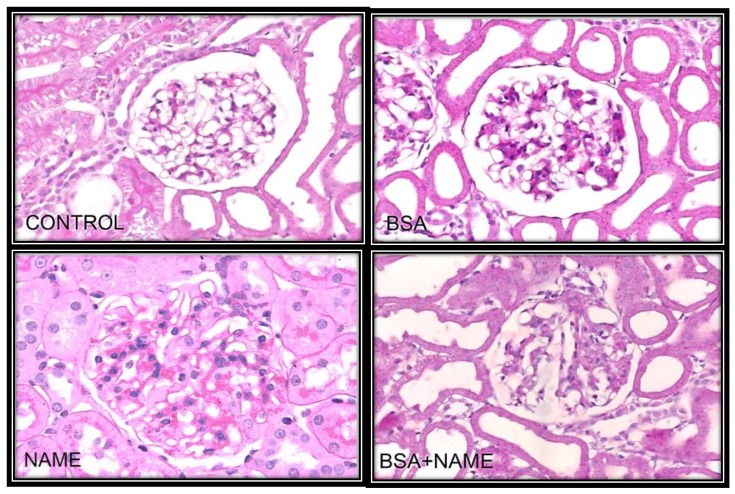
Representative light microscopy in glomeruli of the experimental groups to 40× stain, with hematoxylin and eosin: H-E histological abnormalities present in BSA and BSA + NAME groups, characterized by interstitial fibrosis, sclerosis, retraction of the glomerular tuft that seemed collapsed, and expansion of the mesangium. In the NAME group, hypercellularity was present, with slight expansion of the mesangium.

Figure 10 shows representative photomicrographs of the renal aorta segment in C, BSA, NAME and BSA + NAME groups. The C group did not show histological abnormalities in the kidney. In the NAME group, hypercellularity was present, as was slight expansion of the mesangium. However, histological abnormalities were present in BSA and BSA + NAME groups, characterized by interstitial fibrosis, sclerosis, retraction of the glomerular tuft that seemed collapsed, and expansion of the mesangium.

## 4. Discussion

Due to the function of the kidney in the long-term control of BP, one of the most frequent manifestations of the alteration of the renal regulatory mechanisms is the development of various degrees of SH [19]. In patients with SH, frequent changes in perfusion pressure of the kidney under conditions of maximum stress can aggravate hemodynamic alterations, thus favoring the development of renal failure [20]. Besides, SH alters intrarenal hemodynamics, modifying the intraglomerular pressure-regulating mechanisms, and allows for the progression of glomerulonephritis [21]. This, in turn, can lead to SH with elevation of the glomerular capillary pressure, which may be associated with increased renal blood flow and hyperperfusion of glomeruli [22]. These conditions lead to glomerular collapse, wrinkling and obsolescent glomeruli, which lead to increasing proteinuria and failure in creatinine clearance [23,24]. The lack of eNOS in the glomerular endothelium aggravates this condition in human and experimental glomerulonephritis [25]. However, few studies have addressed the association between glomerulonephritis, SH and the deterioration of the renal vascular function. Therefore, the aim of the present study was to demonstrate the association between ICGN and the SH induced by the administration of L-NAME, and the analysis of renal vascular reactivity. The endothelium is a key factor in vascular reactivity [26]. Our results show that the BSA- and NAME-treated groups exhibit a certain degree of renal endothelial dysfunction because the vasoconstriction and vasodilatation were increased and decreased, respectively. However, with the combination of both treatments, represented by treatment with both BSA and NAME, the damage is more serious, suggesting that the ICGN can be associated with SH induced by L-NAME, and caused by altered renal endothelial function.

## 5. Endothelium

The endothelium is a monolayer of cells that covers the entire arterial tree. It is highly selective and provides a nonthrombogenic surface, actively acting as a sensor of the physical and metabolic state of the cardiovascular system, and controlling the vascular tone and the inflammatory response [27]. For the control of vascular tone, the endothelium synthesizes different vasodilator substances such as nitric oxide (NO) [28], which is the product of the nitric oxide synthases (NOSs). eNOS is constitutively expressed in the kidney by the normal glomerular endothelium and interstitial vessels. Since the amount of NO generated is small (in nmol quantities), its effect is beneficial for vascular reactivity [29], and contributes to the regulation of the glomerular microcirculation by modifying the resistance of the afferent arteriole. Thus, it helps to improve CrCl and mesangial cells, and at the same time, it maintains the antithrombogenic properties of the endothelium [30]. However, when the iNOS pathway is activated, it leads to high NO concentrations (µM or mM amounts), and at these concentrations, NO can be cytotoxic [26]. High NO amounts react with reactive oxygen species, generating peroxynitrite anion formation, protein tyrosine nitration and hydroxyl radical production. This leads to the loss of structural and functional proteins of the intrinsic renal and mesangial cells, favoring inflammation [31].

## 6. Endothelial and Inducible Nitric Oxide Synthases

Furthermore, the results show that the downregulated expression of eNOS and the increase in iNOS expression in the endothelium of the BSA-, NAME- and BSA + NAME-treated groups can favor NO bioavailability, and increased and decreased vasoconstriction and vasodilatation, respectively, which may result in destruction and structural changes of elastic fibers, altering the functions of endothelial cells [32]. Therefore, the endothelial dysfunction observed in this study could be modulated by NO synthesized by iNOS without involvement of the eNOS pathway. The NO increase may have a modulatory effect on the degree of subendothelial matrix integrity, thus contributing to the reduced distensibility of the vessel and altered renal function. In addition, lack of eNOS can induce a loss in the glomerular capacity and in the peritubular capillary endothelium, and exacerbate renal injury in progressive renal disease [32]. eNOS knockout mice develop focal congenital renal abnormalities, including glomerular hypoplasia and tubular cell death, and atubular glomeruli [33,34]. Another study in human glomerulonephritis showed that increased activity of iNOS was associated with inflammatory cytokines such as TNF-α, and this contributed to renal dysfunction [34]. Also, high levels of NO produced by the iNOS pathway in the kidney can favor several forms of glomerulonephritis [35]. In addition, low eNOS expression can lead to SH. Our results show that BP was elevated in BSA- and NAME-treated rats, while in the BSA + NAME-treated group, it was greater than in the last two groups. These results, together with the others, suggest a synergistic effect between the SH caused by the chronic consumption of the NAME and ICGN, which elevated BP in the renal artery and the glomerular capillary pressure. Therefore, a positive feedback system is established between ICGN and SH. Likewise, the L-NAME-induced SH is aggravated in the presence of the ICGN by the participation of the kidney in BP control. This may be associated with alterations of the renal blood flow and hyperperfusion of glomeruli that lead to progressive glomerular damage, which is evidenced by the gradual increase and decrease of proteinuria and CrCl, respectively [36]. In addition, the results of pre-incubation of the renal aortic rings with NAME, together with histological changes, suggest that the NOS pathways are altered, as is the degree of participation in the renal aorta. Furthermore, the effect on the arteries and renal damage in SH induced by L-NAME implies a loss of the renal mass, which worsens the underlying hypertension [37]. This peculiar role of the renal vascular system to potentiate the vicious circle of hypertension is explained by arterial structural or functional narrowing, since it stimulates the renin–angiotensin system, diminishing the renal excretory capacity [38]. Chronic elevation of BP may produce endothelial injury. 

Furthermore, the endothelium is constantly exposed to several stimuli, among which oxidized lipoproteins, friction forces, inflammatory agents, cytokines and free radicals can be highlighted. The repeated stimulation of the endothelium by these factors can lead to endothelial dysfunction in the renal aorta [31], and this can produce structural and functional changes in the smooth muscle cells and elastic fibers of the renal microcirculation [28].

## 7. Elastic Fibers in Renal Aorta

Elastic fibers are the dominant proteins in the arterial extracellular matrix. The elastic fibers, collagen and smooth muscle cells are oriented in a concentric fashion in the aortic medial layer, determining the elastic and dynamic mechanical features of the renal aorta [39]. Our results show that the number of elastic fibers in the aortic medial layer was lower and that the fibers were not continuous but undulating, showing rupture zones and fragmentation in BSA, NAME and BSA + NAME groups. These results may be associated with the lack of relaxation, high vasoconstriction and disorganization that is shown in microphotographs of the aortic renal artery. It has also been described that hypertensive glomerulosclerosis can occur with excessive vasoconstriction with eventual anatomical narrowing of the preglomerular vasculature, leading to glomerular ischemia [40]. Furthermore, the changes in the renal aorta could result from a decrease in the renal function and the consequent increased BP.

## 8. Hypertension and Glomerulonephritis

On the other hand, our direct immunofluorescence results showed IgG-positive immune-complex deposition in BSA and BSA + NAME, and histological abnormalities, in all groups except the C group. This suggests that ICGN happens after the in-situ formation of immune complexes in the glomerular basal membrane. It also appears less frequently after deposition of immune complex in the internal sheet, as has been previously described [4,41]. In addition, selectivity for particle size is provided by the dense sheet of the glomerular basement membrane, which contains small pores that restrict the filtration of macromolecules [29]. Therefore, the molecular characteristics of the antigen and the antibody, or of the immune complexes, are determining factors for the location of immune complexes in the glomerular wall [12]. In addition, the site of deposition or assembly of immune complexes determines the clinical or structural manifestations of the disease. The mesangial cells also participate in the clearance of glomerular immune complexes and other macromolecules [2]. An increase in the expansion of mesangial cells can be the reason why, when mesangial function is impaired, deposits derived from immune complexes persist, and our results show an increase in expansion of the mesangium in the BSA, NAME and BSA + NAME groups. Mesangial immune deposits reflect mesangial dysfunction and contribute to increased permeability of the glomerular capillary [4,8]. This can be associated with SH, which by itself can enhance permeability. Permeability changes finally result in proteinuria [5]. Our results show that proteinuria and CrCl were increased in all groups when compared to those in the C group. In addition, proteinuria is a common feature of declining glomerular filtration rate in patients [40]. SH aggravates nephritic manifestations in spontaneously hypertensive rats with Heymann nephritis, it produces endothelial injury and it enhances the glomerular permeability, finally resulting in proteinuria [41].

## 9. Conclusions

In conclusion, the results suggest that SH induced by L-NAME contributes to the development of progressive renal disease in ICGN. Furthermore, a positive feedback loop between ICGN and hypertension might be established, and the hypertension could be aggravated by the presence of ICGN. Alterations of the vascular reactivity are mediated by the endothelium in the renal aorta. Thus, the endothelium plays a determinant role in the production of vasoactive substances such as NO during this process.

## Figures and Tables

**Table 1:** General characteristics and glomerular filtration index.

Variables	C	BSA	NAME	BSA + NAME
BP (mmHg)	97.32 ± 2.91	126.90 ± 2.24 **	147.07 ± 1.07 ***	173.41 ± 5.36 ***
Proteinuria (mg protein/24 h)	29.89 ± 3.71	50.98 ± 10.06 *	162.66 ± 31.32 ***	178.64 ± 23.90 ***
SCr (µg/dL)	0.68 ± 0.05	0.95 ± 0.10 **	1.18 ± 0.12 **	1.35 ± 0.09 **
UCr (mg/dL)	1.06 ± 0.08	0.52 ± 0.19 *	0.76 ± 0.06 *	0.67 ± 0.36
CrCl (mL/min)	0.0114 ± 0.0015	0.0073 ± 0.0006 *	0.0066 ± 0.0006 ***	0.0051 ± 0.0004 **

Notes: BP = blood pressure; SCr = serum creatinine; UCr = urine creatinine; CrCl = creatinine clearance. Data are means ± SE, *n* = 12 each group. * BSA vs. BSA + NAME *p* = 0.05, ** BSA vs. C and BSA + NAME, and C vs. BSA *p* = 0.03, *** C vs. NAME, and C vs. BSA, C and NAME *p* = 0.001. C: Control; BSA: bovine serum albumin; NAME: an analog of arginine that inhibits NO production.

## References

[1] Stevens P.E., Levin A. (2013). Evaluation and management of chronic kidney disease: Synopsis of the kidney disease: Improving global outcomes 2012 clinical practice guideline. Ann. Intern. Med..

[2] Yang J.J., Jennette J.C., Falk R.J. (1994). Immune complex glomerulonephritis is induced in rats immunized with heterologous myeloperoxidase. Clin. Exp. Immunol..

[3] Jang W.S., Jeong K.H., Moon J.Y., Lee S.H., Cho J.H., Lee T.W., Park Y.K., Cho B.S., Ihm C.G. (2012). Relationship between glomerulomegaly and clinicopathologic findings in IgA nephropathy. Clin. Nephrol..

[4] Gadau J., Peters H., Kastner C., Kühn H., Nieminen-Kelhä M., Khadzhynov D., Krämer S., Castrop H., Bachmann S., Theilig F. (2009). Mechanisms of tubular volume retention in immune-mediated glomerulonephritis. Kidney. Int..

[5] Okuda S., Onoyama K., Fujimi S., Oh Y., Nomoto K., Omae T. (1983). Influence of hypertension on the progression of experimental autologous immune complex nephritis. J. Lab. Clin. Med..

[6] Dixon F.J., Wilson C.B., Marquardt H. (1971). Experimental immunologic glomerulonephritis. Adv. Nephrol. Necker. Hosp..

[7] Yamamoto K., Oite T., Kihara I., Shimizu F. (1984). Experimental glomerulonephritis induced by human IgG in rats. Clin. Exp. Immunol..

[8] Woitas R.P., Morioka T. (1996). Influence of isoelectric point on glomerular deposition of antibodies and immune complexes. Nephron.

[9] Pirkle J.L., Freedman B.I. (2013). Hypertension and chronic kidney disease: Controversies in pathogenesis and treatment. Minerva. Urol. Nefrol..

[10] Sánchez T.G., Baños G. (2004). Hipertensión Arterial: Fisiopatología.

[11] Iversen B.M., Amann K., Kvam F.I., Wang X., Ofstad J. (1998). Increased glomerular capillary pressure and size mediate glomerulosclerosis in SHR juxtamedullary cortex. Am. J. Physiol..

[12] Chen Y., Tang Z., Yang G., Shen S., Yu Y., Zeng C., Chen H., Liu Z.H., Li L.S. (2005). Malignant hypertension in patients with idiopathic IgA nephropathy. Kidney Blood Press. Res..

[13] Ihm C.G. (2015). Hypertension in Chronic Glomerulonephritis. Electr. Blood Press..

[14] Pérez-Torres I., Roque P., El Hafidi M., Diaz-Diaz E., Baños G. (2009). Association of renal damage and oxidative stress in a rat model of metabolic syndrome. Influence of gender. Free. Radic. Res..

[15] BenGershom E. (1975). Screening for albuminuria: A case for estimation of albumin in urine. Clin. Chem..

[16] Perrone R.D., Madias N.E., Levey A.S. (1992). Serum creatinine as an index of renal function: New insights into old concepts. Clin. Chem..

[17] Luna G.L. (1967). Histopathology Laboratories, 3a Edition, Armed Forces Institute of Pathology Washington, D.C. Eds.

[18] Perez-Torres I., El Hafidi M., Carvajal K., Baños G. (2009). Castration modifies aortic vasoreactivity and serum fatty acids in a sucrose-fed rat model of metabolic syndrome. Heart Vessel..

[19] Puar T.H., Mok Y., Debajyoti R., Khoo J., How C.H., Ng A.K. (2016). Secondary hypertension in adults. Singap. Med. J..

[20] Freedman B.I., Sedor J.R. (2008). Hypertension-associated kidney disease: Perhaps no more. J. Am. Soc. Nephrol..

[21] Anjum S., Muzaale A.D., Massie A.B., Bae S., Luo X., Grams M.E., Lentine K.L., Garg A.X., Segev D.L. (2016). Patterns of End-Stage Renal Disease Caused by Diabetes, Hypertension, and Glomerulonephritis in Live Kidney Donors. Am. J. Trans..

[22] Stein H.D., Sterzel R.B., Hunt J.D., Pabst R., Kashgarian M. (1986). No aggravation of the course of experimental glomerulonephritis in spontaneously hypertensive rats. Am. J. Pathol..

[23] Luke R.G. (2006). Hypertensive nephrosclerosis. Kidney Int..

[24] Hill G.S., Heudes D., Jacquot C., Gauthier E., Bariéty J. (2006). Morphometric evidence for impairment of renal autoregulation in advanced essential hypertension. Kidney Int..

[25] Heeringa P., van Goor H., Itoh-Lindstrom Y., Maeda N., Falk R.J., Assmann K.J., Kallenberg C.G., Jennette J.C. (2000). Lack of endothelial nitric oxide synthase aggravates murine accelerated anti-glomerular basement membrane glomerulonephritis. Am. J. Pathol..

[26] Meenakshi S.R., Agarwal R. (2013). Nitric oxide levels in patients with chronic renal disease. J. Clin. Diagn. Res..

[27] Pérez-Torres I., El Hafidi M., Infante O., Baños G. (2008). Effects of sex hormone levels on aortic vascular reactivity and variables associated with the metabolic syndrome in sucrose-fed female rats. Can. J. Physiol. Pharmacol..

[28] Soto M.E., Iturriaga H.V., Guarner-Lans V., Zuñiga-Muñoz A., Aranda F.A., Velázquez E.R., Pérez-Torres I. (2016). Participation of oleic acid in the formation of the aortic aneurysm in Marfan syndrome patients. Prostaglandins. Other Lipid Med..

[29] Cook H.T., Sullivan R. (1991). Glomerular nitrite synthesis in in situ immune complex glomerulonephritis in the rat. Am. J. Pathol..

[30] Furusu A., Miyazaki M., Abe K., Tsukasaki S., Shioshita K., Sasaki O., Miyazaki K., Ozono Y., Koji T., Harada T. (1998). Expression of endothelial and inducible nitric oxide synthase in human glomerulonephritis. Kidney Int..

[31] Nakagawa T., Tanabe K., Croker B.P., Johnson R.J., Grant M.B., Kosugi T., Li Q. (2011). Endothelial dysfunction as a potential contributor in diabetic nephropathy. Nat. Rev. Nephrol..

[32] Forbes M.S., Thornhill B.A., Park M.H., Chevalier R.L. (2007). Lack of endothelial nitric-oxide synthase leads to progressive focal renal injury. Am. J. Pathol..

[33] Kashem A., Endoh. M., Yano N., Yamauchi F., Nomoto Y., Sakai H. (1996). Expression of inducible-NOS in human glomerulonephritis: The possible source is infiltrating monocytes/macrophages. Kidney Int..

[34] Jansen A., Cook T., Taylor G.M., Largen P., Riveros-Moreno V., Moncada S., Cattell V. (1994). Induction of nitric oxide synthase in rat immune complex glomerulonephritis. Kidney Int..

[35] Martínez-Maldonado M. (1998). Hypertension in end-stage renal disease. Kidney Int. Suppl..

[36] Scarpelli P.T., Gallo M., De Cesaris F., Chiari G., Dedola G., Cappeli S., Becucci A., Becherelli P., Tosi B., Fanetti C. (2002). Continuing follow-up of malignant hypertension. J. Nephrol..

[37] Kuijpers M.H., Gruys E. (1984). Spontaneous hypertension and hypertensive renal disease in the fawn-hooded rat. Br. J. Exp. Pathol..

[38] Ofstad J., Iversen B.M. (2005). Glomerular and tubular damage in normotensive and hypertensive rats. Am. J. Physiol. Renal. Physiol..

[39] Soto M.E., Guarner-Lans V., Herrera-Morales K.Y., Pérez-Torres I. (2018). Participation of arachidonic acid metabolism in the aortic aneurysm formation in patients with marfan syndrome. Front. Physiol..

[40] Nakayama T., Sato W., Kosugi T., Zhang L., Campbell-Thompson M., Yoshimura A., Croker B.P., Johnson R.J., Nakagawa T. (2009). Endothelial injury due to eNOS deficiency accelerates the progression of chronic renal disease in the mouse. Am. J. Phys. Ren. Physiol..

[41] Scarpelli P.T., Gallo M., De Cesaris F., Chiari G., Dedola G., Cappeli S., Becucci A., Becherelli P., Tosi B., Fanetti C. (2012). Intrinsic proinflammatory signaling in podocytes contributes to podocyte damage and prolonged proteinuria. Am. J. Physiol. Ren. Physiol..

